# Iterative Learning Control for Motion Trajectory Tracking of a Circular Soft Crawling Robot

**DOI:** 10.3389/frobt.2019.00113

**Published:** 2019-11-12

**Authors:** Haozhen Chi, Xuefang Li, Wenyu Liang, Jiawei Cao, Qinyuan Ren

**Affiliations:** ^1^College of Control Science and Engineering, Zhejiang University, Hangzhou, China; ^2^Department of Electrical and Electronic Engineering, Imperial College London, London, United Kingdom; ^3^Department of Electrical and Computing Engineering, National University of Singapore, Singapore, Singapore; ^4^Temasek Laboratories, National University of Singapore, Singapore, Singapore

**Keywords:** ILC, soft crawling robot, dielectric elastomer actuator, electro-adhesion actuator, knowledge-guided data-driven modeling

## Abstract

Soft robots have recently received much attention with their infinite degrees of freedoms and continuously deformable structures, which allow them to adapt well to the unstructured environment. A new type of soft actuator, namely, dielectric elastomer actuator (DEA) which has several excellent properties such as large deformation and high energy density is investigated in this study. Furthermore, a DEA-based soft robot is designed and developed. Due to the difficulty of accurate modeling caused by nonlinear electromechanical coupling and viscoelasticity, the iterative learning control (ILC) method is employed for the motion trajectory tracking with an uncertain model of the DEA. A *D*^2^ type ILC algorithm is proposed for the task. Furthermore, a knowledge-based model framework with kinematic analysis is explored to prove the convergence of the proposed ILC. Finally, both simulations and experiments are conducted to demonstrate the effectiveness of the ILC, which results show that excellent tracking performance can be achieved by the soft crawling robot.

## 1. Introduction

Currently, there is a great interest in using the soft robots for practical applications. In contrast to conventional rigid (hard) robots, soft robots are constructed with compliant materials that can be stretched, bent and twisted in new ways. Thus, soft robots possess the potential demonstrating unprecedented adaptation, sensitivity and agility.

Soft actuators play the key role in the soft robots. In previous studies, many soft actuators have been investigated, such as pneumatic muscle actuator (PMA) (Andrikopoulos et al., 2011; Rolf and Steil, 2012; Onal and Rus, 2013), shape memory alloy (SMA) (Koh and Cho, 2009; Yuk et al., 2011) and electroactive polymer (EAP) (Yeom and Oh, 2009; Lau et al., 2014; Godaba et al., 2017). Among these actuators, dielectric elastomer actuators (DEAs) which is one of the EAPs stand out in robotic applications due to their special properties including large deformation, fast response, high energy density, low noise and biological muscle similarities. The muscle-like properties and relatively simple actuation method of DEAs have contributed to developing several bio-inspired soft robots, such as worm-like crawling robots (Shian et al., 2015; Mihai Duduta and Wood, 2017; Cao et al., 2018a), underwater robots (Guo et al., 2012; Li et al., 2017) and human-like robots (Carpi and Rossi, 2005; Liu et al., 2008; Wang and Zhu, 2016). There are two notable work Qin et al. (2018) and Gu et al. (2018) should be pointed out. The work presented in Qin et al. (2018) focuses on the robot prototype with its motion, while the work presented in Gu et al. (2018) emphasizes more on the adhesion method.

The circular soft crawling robot proposed in Qin et al. (2018) is investigated in this study, which is a promising platforms for practical application or scientific research. This robot uses the DE materials as the robot body, which can not only provide large strain with high deformation, but also realize the similar properties of the biological muscles owing to their similarity. Besides that, four electroadhesion actuators are employed as the robot feet to provide adaptive and low-power bonding adhesion action on the ground surface, which has been described in Gu et al. (2018). In Gu et al. (2018), gave a detailed description of electroadhesion with corresponding experimental demonstrations, which shows the advantages of electroadhesion method. By comparing to the traditional adhesion mechanisms such as suction cup (Longo and Muscato, 2006), adhesive (Murphy and Sitti, 2007; Sangbae et al., 2008), magnetic adsorption (Shen et al., 2014), etc., electroadhesion has two significant advantages: (i) simple structure and easy to be manufactured; (ii) not subjected to the effect of the working surface, only requires the working surface can generate induced charges. However, due to the feature of the electrostatic force and the strength limitation of the applied voltage (electrostatic breakdown), the adhesion force caused by the electroadhesion is much smaller than the vacuum suction force or the electromagnetic force. Nevertheless, in this study, due to the light weight of the robot itself, the required frictional force is not large during its movement process. Therefore, the defect of low adhesion force can be ignored in this study, which makes electroadhesion an appropriate way. Similarly, the electroadhesion method also enables the entire robot to be fully electric-powered, which leads to a consistency with the energy source. The use of DEA and electroadhesion actuators render the robot to be light-weight and fast-response. Moreover, inspired by the bionics, the robot can achieve stable locomotion through alternating expansion/contraction of the body as well as adhesion/release of the feet.

Although the DEA-based soft robots exhibit muscle-like motions in various environments, the studies presented in Qin et al. (2018) and Gu et al. (2018) mainly focus on the robot modality which is based on the open-loop control. Only a few studies on the robot control like (Cao et al., 2018a,b) are available. In addition, there is a major practical challenge lies in the control issue due to the electromechanical coupling and viscoelasticity of the DE materials. Due to the significance of the “control” to the whole system, this study mainly aims to explore the motion control issue of the soft crawling robot for the better performance and greater application value.

Currently, the researches on both open-loop and closed-loop control are extremely limited. In Gu et al. (2015), a feed-forward control approach is proposed for a planar DEA. However, this control approach can not adjust the performance of the control outputs due to the lack of feedback information. In order to enhance the robustness of the control system, feedback control schemes have been adopted in several studies, the notable examples include the classical proportional-integral-derivative (PID) control scheme presented in Rizzello et al. (2015) and Cao et al. (2018a) and the cerebellum-inspired adaptive controller proposed in Wilson et al. (2016); Cao et al. (2018b). It should be pointed out that these previous researches on controlling DEA is primarily restricted with isolated actuators in simple geometries, and the control effect depends on model accuracy due to the model-based property (Rivera et al., 1986; Hong et al., 2014). So far, although various soft robots driven by DEA have been developed (Godaba et al., 2017; Tang et al., 2017), few studies have focused on the motion control of the DEA-based soft robots, which greatly hinders these soft robots from practical application.

The main objective of this work is to address the precise displacement tracking problem of a circular soft crawling robot via iterative learning control (ILC), which is essential to both motion control and motion planning. To our best knowledge, this is a pioneering work in the field of DEA-based soft robot with ILC, which will demonstrate the effectiveness of ILC in the motion control of DEA-based circular crawling robot. ILC, as an effective control strategy, is designed to improve the current performance of uncertain systems by fully utilizing the past control experience. Specifically, ILC is developed for systems that are able to complete some tasks over a finite time interval and perform them repeatedly. In such systems, the input and output information of past cycles, as well as the tracking objective, are used to formulate the input signal for the next iteration, hence the tracking performance could be improved iteratively. By comparing to traditional control techniques, such as PID control and fuzzy logic control, there are a number of distinct features about ILC. Primarily, ILC is designed to handle repetitive tasks. The traditional control methods cannot deal with or take advantage of the periodic nature. Under a repeatable control environment, repeating the same feedback would yield the same control performance. By incorporating learning, ILC is able to improve the control performance iteratively. Apart from this, the control objective is different. ILC tries to achieve perfect tracking during the whole operation interval, whereas most other control methods usually achieve asymptotic error convergence in the time domain. Last but not least, ILC is a partially model-free control method (Tan, 2003). As long as an appropriate learning gain is chosen, the excellent tracking performance can be achieved even when the system parameters are unknown. There is one typical situation for its application for explaining the advantages: when setting the robot in a complex environment where each area has its own terrain features, its optimal parameters are undoubtedly different in each area. In this situation, because that the ILC can adaptively adjust the parameters during the iteration, only a single set of parameters needs to be determined when adapted to the complex environment, without setting separate parameters for each type of environmental information, which really improves robot's applicability and environment adaptability.

Simultaneously, the motivation of adopting ILC for the soft robot control also comes from three aspects. Firstly, due to the action principle of the DEA, generating a desired sequence motion trajectory is essential to actuate the robot in a cluttered environment. In addition, since the DEA only performs open-loop control in each action cycle, for the sake of improving the performance of the robot's motion trajectory tracking, the input and output information of the DEA control system in past cycles is generally used to formulate the input signal for the next iteration which is totally repetitive tasks. Secondly, as pointed out by Cao et al. (2018a,b), one of the main reasons that deters us from controlling DEA-based soft robots is its difficulty of obtaining accurate models. Therefore, the model-free control methods can greatly reduce the workload, not to mention its excellent tracking performances. Thirdly, it has been proved that ILC is easy to be implemented. After first proposed by Arimoto et al. (2010), ILC has been extensively studied with significant progress in theory (Bien and Xu, 1998; Bristow et al., 2006), as well as wildly applied in practice, such as industrial robots (Barton and Alleyne, 2010), mobile robots (Ostafew et al., 2013), manipulators (Tayebi and Islam, 2006; Cong et al., 2017), electronic motors (Panda et al., 2008; Mohammadpour et al., 2013), as well as motion control of robotic fish (Li et al., 2016), etc.

The main contribution for this paper is that the model-free iterative learning controller is adopted to eliminated the demand of model accuracy which simplifies the difficult modeling process, and several simulations with relevant experiments are conducted to prove its effectiveness. The rest of this paper is organized as follows. Section 2 introduces the robot platform and its kinematics analysis. In section 3, the dynamical model of the soft robot is built. Section 4 presents the ILC design and its convergence analysis. Furthermore, the efficiency of the proposed ILC scheme is verified by both simulations and experiments in section 5. Finally, the conclusion is given in section 6.

## 2. Soft Robot Design

**Figure 1:**
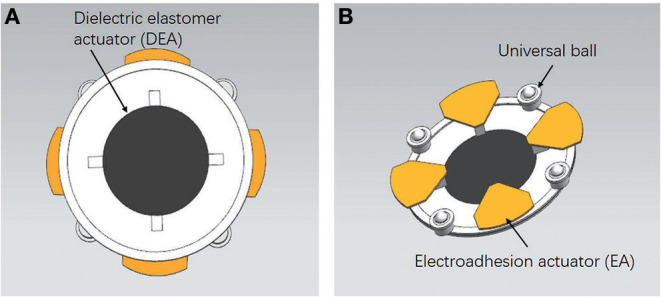
The soft mobile robot prototype. **(A)** Top view of the soft robot. **(B)** The bottom structure of the soft robot.

A soft crawling robot is designed and fabricated as illustrated in Figure 1. The robot mainly consists of a circular DEA and four electroadhesion actuators, which work together to achieve flexible 2D planar crawling. In addition, four passive omnidirectional wheels are mounted on the robot to reduce the moving frictional resistance.

### 2.1. Robot Body

**Figure 2:**
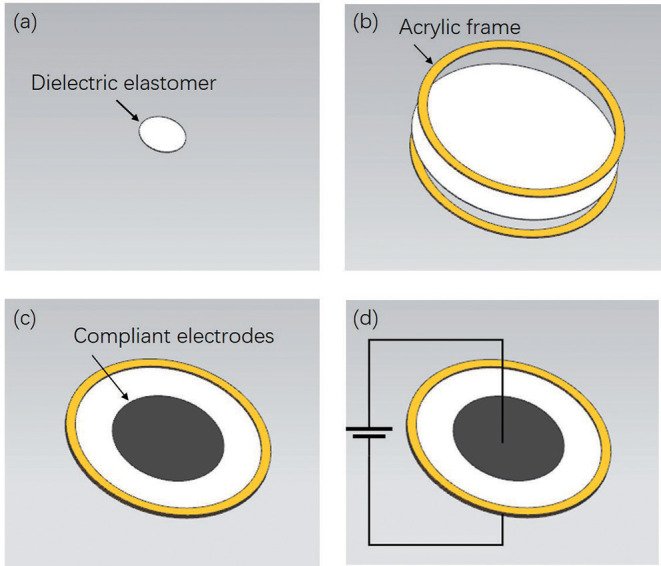
The fabrication process of the robot body. **(a)** Initial state of the Di-electric elastomer film. **(b)** Pre-stretching processing. **(c)** Coating the electrodes. **(d)** Generating the deformation.

Figure 2 illusitrates the manufacturing process of the robot body. The DEA is essentially a VHB4910 membrane sandwiched between compliant electrodes. Initially, the membrane is subjected to 4 × 4 equal-biaxial pre-stretching with the radius of 100 mm under the constraint of two annular Acrylic frames. Two compliant electrodes are smeared evenly on both surfaces of the membrane to obtain a conductive region. When the high voltage applies, the membrane will result in a thickness reduction and area expansion caused by voltage-induced Maxwell stress (Zhigang, 2010). Moreover, the membrane will restore to its original state under the action of internal elastic contraction force when the voltage is off.

With regard to the specific actuation principle, the electro-mechanical response of DEA relies primarily on the Maxwell force and blocking force, which determine its nature of stretching and contraction (Zhao et al., 2018).

**Figure 3:**
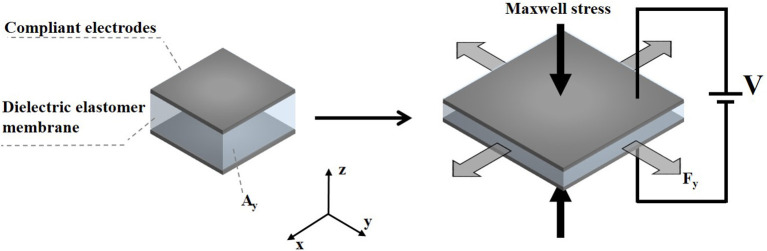
The actuation principle of a DEA. V is the voltage applied across the membrane, *A*_*y*_ indicates the cross-sectional area in the y direction and *F*_*y*_ presents the blocking force in the y direction.

As shown in Figure 3, the Maxwell stress perpendicular to the surface of the DE membrane is generally considered to describe the mechanical response of the electrical stimulation, which is essentially the electric field force in the electric field formed between the compliant electrodes on both sides of the membrane. Thus, the specific magnitude of the Maxwell stress can be described by:

(1) $$\begin{array}{l} \sigma_{M}=\varepsilon_{0}\varepsilon_{r}E^{2}=\varepsilon_{0}\varepsilon_{r}{\left(V/d\right)}^{2}, \end{array}$$

where ε_0_ represents the vacuum dielectric constant, ε_*r*_ represents the relative dielectric constant of the material, *V* is the voltage applied across the membrane, and *d* is the distance between the two compliant electrodes.

The blocking force refers to the force required to restore the fully energized actuator in the lateral dimension, which is a resistance that needs to be overcome during the actuation. It can be calculated by:

(2) $$\begin{array}{l} \sigma_{block}=F_{y}/A_{y}=\left(x_{0}y_{0}z_{0}\varepsilon_{0}\varepsilon_{r}E^{2}\right)/\left(yA_{y}\right), \end{array}$$

where *A*_*y*_ indicates the cross-sectional area in the y direction.

### 2.2. Robot Feet

**Figure 4:**
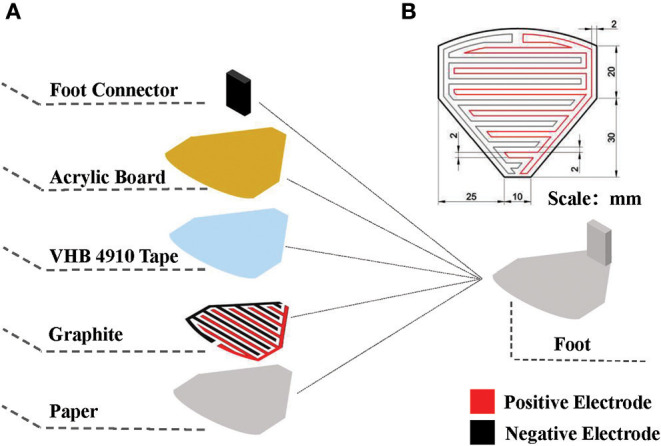
Schematic of the robot foot. **(A)** Exploded view, **(B)** dimensions of the electrode pattern.

Figure 4 illustrates the composition of an electroadhesion actuator. The electrode pattern with the dimensions shown in Figure 4B is first designed and printed on a ordinary paper. Later on, the area enclosed by the lines is scribbled with graphite by a 2B pencil for creating a conductive region. Finally, pieces of VHB4910 membranes are used to bond the conductive layer to the Acrylic board and its foot connector. It is worth mentioning that the insulation property of the VHB layer also helps to prevent the electrodes from short-circuit via external substances.

**Figure 5:**
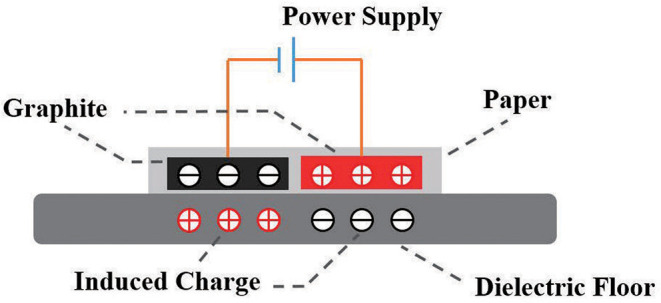
The working principle of a EA.

As shown in Figure 5, when the electroadhesion actuator is subjected to a high voltage, the two electrodes accumulate separately positive and negative charges. Furthermore, the electric field generated by the charged electrodes causes opposite induced charges on the substrate, thereby creating an electroadhesion force (electrostatic attraction force) between the actuator and the substrate (Shintake et al., 2016). Besides, a paper layer is served as an insulating layer to prevent the inductive charges on the substrate from neutralizing the charges on the electrodes. After the absence of a voltage, the charges on the electrodes disappear as well as the electroadhesion force, thereby leading to a reversible adhesion.

### 2.3. Locomotion

**Figure 6:**
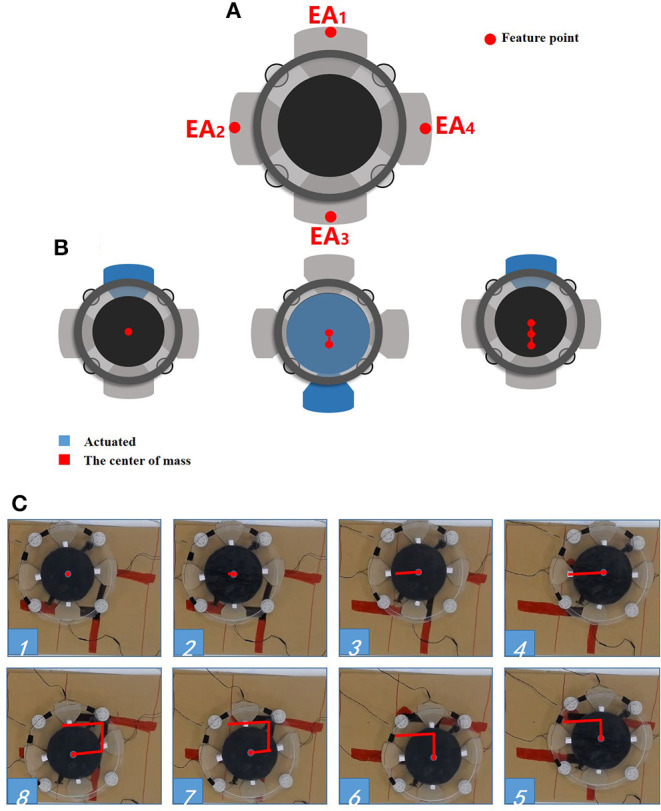
The locomotion of the soft robot. **(A)** The feet number; **(B)** the periodic movement; **(C)** the direct tracking experiment.

Figure 6A shows the labeling of the four electroadhesion actuators (EAs). Due to the interaction of the four feet, the robot is able to achieve 2D motion. Figure 6B schematically demonstrates a periodic single-dimensional movement of this soft robot by the following actuation sequence.

At the first step of the loop, only the foot *EA*_1_ is subjected to the voltage while all the others are not powered. thus the *EA*_1_ adheres to the substrate. At the second step, the *EA*_3_ and the DEA are activated simultaneously, without powering on the remaining EAs. As a result, *EA*_3_ produces an electroadhesion force to fix itself on the substrate, and the robot body extends under the drive of DEA's expanding. Thus, the *EA*_1_ is pushed forward. The third steps repeats the first step with the same voltage signals, causing the *EA*_1_ to be attached on the substrate. Without the voltage induced Maxwell stress, the DEA will return to its original state. Consequently, the *EA*_3_ is pulled toward the DEA's center and hence the entire robot moves forward. Therefore, by repeating the above actuation sequence, the soft robot will gradually move forward cycle by cycle. By reversing the actuation sequence of *EA*_1_ and *EA*_3_, the soft robot can move backwards.

Benefit from the isotropy brought about by the circular structure, the soft robot is able to achieve omnidirectional motion easily which is particularly suitable for unstructured environments.

Figure 6C shows a simple trajectory tracking motion in 2D plane. During the test, an external camera is used to obtain the trajectory information (the “red” line in the figure) as the feedback signals to realize the C-shaped trajectory tracking. As can be seen, the soft robot is able to track the C-shaped trajectory, which shows that the soft robot can achieve omnidirectional motion by using the appropriate sequences of the actuation.

## 3. Knowledge-Based Modeling

Modeling the dynamics of the robot body is critical to the motion controller design. Due to the difficulty of the soft robot modeling, a data-driven ILC method is investigated in this study. Moreover, a knowledge-based model framework is used to build a simplified dynamic model of the DEA that can be used to export the generalization of the proposed ILC scheme.

It should be explained here that the modeling is only for DEA, not including EA, and so as the control scheme mentioned in section 4. This does not mean that the EAs is ignorable, but just because the actuation for them is relatively simple which does not require a complicated processing. Furthermore,the main motion of the robot is generated by the DEA. The EAs are play roles of alternating adhesive via sequential control. The main topic of the motion control of this study focuses on the motion trajectory tracking. Hence, the paper mainly focuses on controller design for the DEA. Thus, for its simple actuation process, we will not elaborate the EA on its modeling and control.

**Figure 7:**
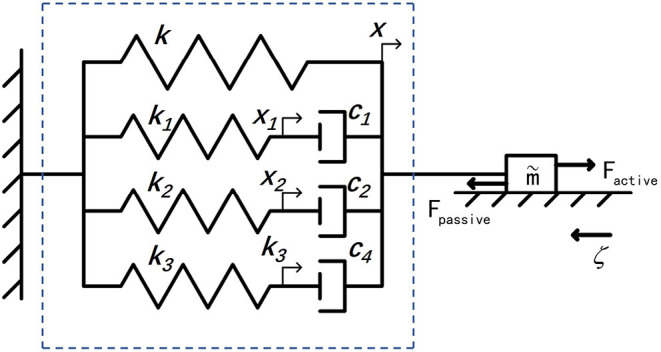
The dynamic model of the DEA, where *k*, *x* is the stiffness and the displacement of a linear spring, respectively, *x*_*h*_ presents the spring deformation, *k*_*h*_ presents the spring stiffness, and *c*_*h*_ shows the viscous friction coefficient. Otherwise, *F*_*active*_ is the active driving force, *F*_*passive*_ means the whole passive resistance generated by the model, ζ illustrate the frictional resistance force during the motion, and $\tilde{m}$ is the equivalent mass of the whole system.

Consider that only the change of displacement and the driving force between the EA feet during the crawling of this soft robot are focused on in this work, the junctions between the DEA and the EA feet are selected as the feature points (see Figure 6A). Moreover, the change of displacement between the corresponding feature points is defined as the robot displacement. In view of the similarity between DE materials and biological muscles in terms of viscoelasticity, a simplified spring-dashpot model mentioned in Gu et al. (2017) is employed to describe the single-dimensional physical properties of the DEA. Figure 7 shows a schematic representation of a dynamic model based on a series of spring-dashpot sets. According to the previous work pointed out in Cao et al. (2018c), a relatively simple third-order system has been used to describe this system. The static tensile force is described by a linear spring with a stiffness *k*. The spring-dashpot parameters include spring deformation *x*_*h*_, spring stiffness *k*_*h*_ and viscous friction coefficient *c*_*h*_ (*h* = 1, 2, 3). The model uncertainties mainly come from the equivalent mass $\tilde{m}$ of the system and the frictional resistance force ζ during the motion. Therefore, the dynamic equations can be given by

(3a) $$\begin{array}{l} \tilde{m}\ddot{x}=F_{passive}+F_{active}-\zeta sign\left(\dot{x}\right), \end{array}$$

(3b) $$\begin{array}{l} F_{passive}=-kx-\overset{3}{\underset{h=1}{\sum }}k_{h}x_{h}, \end{array}$$

(3c) $$\begin{array}{l} k_{h}x_{h}=c_{h}\left(\dot{x}-{\dot{x}}_{h}\right),h=1,2,3. \end{array}$$

The voltage induced compression force *F*_*active*_ can be considered as the product of an equivalent Maxwell stress and an equivalent cross-sectional area of the actuator associated with the deformation. Since the Maxwell stress is proportional to the square of the electric field, *F*_*active*_ can be written as

(4) $$\begin{array}{l} F_{active}=g\left(x\right)V^{2}, \end{array}$$

where *V* is the applied voltage and *g*(*x*) is a function of the change of displacement *x* related to the deformation. Furthermore, it is found that *g*(*x*) is a linear function via the experimental analysis, thus (4) can be rewritten as

(5) $$\begin{array}{l} F_{active}=\left(\alpha x+\beta \right)V^{2}, \end{array}$$

where α and β are the coefficients of the first-order polynomial, the units are *N*·*cm*^−^1·*kV*^−2^ and *N*·*kV*^−2^. The specific value will be obtained by the experiment.

By converting the above differential equations into a state-space model, we can have

(6a) $$\begin{array}{l} \dot{X}=AX+Bu, \end{array}$$

(6b) $$\begin{array}{l} y=CX, \end{array}$$

where

(7) $$\begin{array}{l} u=\frac{F_{active}+\tilde{f}}{\tilde{m}},F_{active}\geq 0, \end{array}$$

(8) $$\begin{array}{l} \tilde{f}=-\zeta sign\left(\dot{x}\right), \end{array}$$

(9) $$\begin{array}{l} X={\left[\begin{array}{ccccc} x_{1} & x_{2} & x_{3} & x & \dot{x} \end{array}\right]}^{T}, \end{array}$$

and

(10) $$\begin{array}{l} A=\left[\begin{array}{ccccc} -k_{1}/c_{1} & 0 & 0 & 0 & 1\\ 0 & -k_{2}/c_{2} & 0 & 0 & 1\\ 0 & 0 & -k_{3}/c_{3} & 0 & 1\\ 0 & 0 & 0 & 0 & 1\\ -k_{1}/\tilde{m} & -k_{2}/\tilde{m} & -k_{3}/\tilde{m} & -k/\tilde{m} & 0 \end{array}\right], \end{array}$$

(11) $$\begin{array}{l} B={\left[\begin{array}{ccccc} 0 & 0 & 0 & 0 & 1 \end{array}\right]}^{T}, \end{array}$$

(12) $$\begin{array}{l} C=\left[\begin{array}{ccccc} 0 & 0 & 0 & 1 & 0 \end{array}\right]. \end{array}$$

Among the model parameters, the stiffness *k* mainly describes the elastic properties under static conditions, α and β mainly represent the electromechanical coupling relationship, $\tilde{m}$ reflects the mass of the robot platform itself, and ζ is mainly dependent on static properties such as friction coefficient when considering only low-speed motion. The spring stiffness *k*_*h*_ and the viscous friction coefficient *c*_*h*_ determine the robot's dynamic characteristics, reflecting the viscoelasticity and creep property.

## 4. Controller Design

In this work, the control objective is to drive the robot to follow a predefined target trajectory. To this end, the ILC scheme is designed. On the basis of the analysis presented in the previous section, the system dynamics of the robot in iteration domain is

(13a) $$\begin{array}{l} X_{k}=AX_{k}+Bu_{k}, \end{array}$$

(13b) $$\begin{array}{l} y_{k}=CX_{k}, \end{array}$$

where *k* is the iteration index and

(14) $$\begin{array}{l} u_{k}≜\frac{V_{k}^{2}\left(\alpha x_{k}+\beta \right)-\zeta \text{ sign}\left({\dot{x}}_{k}\right)}{\tilde{m}} \end{array}$$

is a virtual control input to the system (the actual control input to the robot is the voltage *V*_*k*_).

To facilitate the convergence analysis, it is assumed that the target trajectory *y*_*d*_ is generated by the following dynamical system

(15a) $$\begin{array}{l} X_{d}=AX_{d}+Bu_{d}, \end{array}$$

(15b) $$\begin{array}{l} y_{d}=CX_{d}. \end{array}$$

This is a common assumption in the area of ILC. Then, the control objective is to find a sequence of *u*_*k*_ such that the system output *y*_*k*_ can track the desired target *y*_*d*_ as close as possible.

### 4.1. ILC Design

From the modeling part, it is clear that *CB* = 0, i.e., consequently the relative degree of the system is higher than one, which motivates us to design a higher order ILC scheme. By noting the property *CAB* = 1, a *D*^2^ type ILC law is proposed to the first-order controller (only the last iteration information is referenced in the scheme) (Yin et al., 2009)

(16) $$\begin{array}{l} V_{k}=\sqrt{\frac{u_{k}\tilde{m}+\zeta \text{ sign}\left({\dot{x}}_{k}\right)}{\alpha x_{k}+\beta }}, \end{array}$$

(17) $$\begin{array}{l} u_{k+1}=u_{k}+\gamma {\ddot{e}}_{k}. \end{array}$$

where γ means the learning gain that determines the speed and effect of the iteration, as the only parameter need to be set in the whole process.

The convergence of the proposed controller is summarized in Theorem 1.

**Theorem 1**. *For the system (13), associated with the ILC law (17), the tracking error *e*_*k*_ will converge to zero as *k* → ∞, if γ* ∈ (0, 2).

***Proof:*** Denote *e*_*k*_ ≜ *y*_*d*_ − *y*_*k*_, Δ*X*_*k*_ ≜ *X*_*d*_ − *X*_*k*_, Δ*u*_*k*_ ≜ *u*_*d*_ − *u*_*k*_. By considering the ILC law (17) and the definition of Δ*u*_*k*_, it gives

(18) $$\begin{array}{l} \Delta u_{k+1}=u_{d}-u_{k+1}\\ \;=u_{d}-\left(u_{k}+\gamma {\ddot{e}}_{k}\right)\\ \;=\Delta u_{k}-\gamma {\ddot{e}}_{k}. \end{array}$$

Since Δ*Ẋ*_*k*_ = *A*Δ*X*_*k*_ + *B*Δ*u*_*k*_ and *CB* = 0, we can obtain

(19) $$\begin{array}{l} {\dot{e}}_{k}={\dot{y}}_{d}-{\dot{y}}_{k}\\ \;=C\Delta {\dot{X}}_{k}\\ \;=C\left(A\Delta X_{k}+B\Delta u_{k}\right)\\ \;=CA\Delta X_{k} \end{array}$$

which therefore implies that

(20) $$\begin{array}{l} {\ddot{e}}_{k}=CA\Delta {\dot{X}}_{k}\\ \;=CA^{2}\Delta X_{k}+CAB\Delta u_{k}. \end{array}$$

By substituting (20) into (18), there has

(21) $$\begin{array}{l} \Delta u_{k+1}=\left(1-\gamma CAB\right)\Delta u_{k}-\gamma CA^{2}\Delta X_{k}\\ \;=\left(1-\gamma \right)\Delta u_{k}-\gamma CA^{2}\Delta X_{k} \end{array}$$

because *CAB* = 1.

As the solution of (13a) is

(22) $$\begin{array}{l} X_{k}\left(t\right)=e^{At}X_{k}\left(0\right)+\int_{0}^{t}e^{A\left(t-\tau \right)}Bu_{k}\left(\tau \right)d\tau , \end{array}$$

then we have

(23) $$\begin{array}{l} \Delta X_{k}\left(t\right)=\int_{0}^{t}e^{A\left(t-\tau \right)}B\Delta u_{k}\left(\tau \right)d\tau , \end{array}$$

provided that *X*_*d*_(0) = *X*_*k*_(0). Taking norm on both sides of (23) yields

(24) $$\begin{array}{l} ∥\Delta X_{k}\left(t\right)∥=\int_{0}^{t}e^{a\left(t-\tau \right)}b∥\Delta u_{k}\left(\tau \right)∥d\tau , \end{array}$$

where *a* ≥ ||*A*|| and *b* ≥ ||*B*||. Define $∥g\left(t\right)∥{}_{\lambda }≜\sup_{t\in \left[0,T\right]}e^{-\lambda t}∥g\left(t\right)∥$, then (24) gives

(25) $$\begin{array}{l} ∥\Delta X_{k}\left(t\right)∥\leq be^{at}\int_{0}^{t}e^{\left(\lambda -a\right)\tau }d\tau ∥\Delta u_{k}\left(t\right)∥{}_{\lambda }\\ \;=b\frac{e^{\lambda t}-e^{at}}{\lambda -a}∥\Delta u_{k}\left(t\right)∥{}_{\lambda }. \end{array}$$

Hence we have

(26) $$\begin{array}{l} ∥\Delta X_{k}\left(t\right)∥{}_{\lambda }=\underset{t\in \left[0,T\right]}{\sup}e^{-\lambda t}∥\Delta X_{k}\left(t\right)∥\\ \;\leq b\underset{t\in \left[0,T\right]}{\sup}\frac{1-e^{-\left(\lambda -a\right)t}}{\lambda -a}∥\Delta u_{k}\left(t\right)∥{}_{\lambda }\\ \;\leq b\frac{1-e^{-\left(\lambda -a\right)T}}{\lambda -a}∥\Delta u_{k}\left(t\right)∥{}_{\lambda }\\ \;≜O\left(\lambda^{-1}\right)∥\Delta u_{k}\left(t\right)∥{}_{\lambda } \end{array}$$

for a sufficiently large λ > *a*.

By taking the λ-norm on both sides of (21) and applying (26), there has

(27) $$\begin{array}{l} ∥\Delta u_{k+1}∥{}_{\lambda }=\left(|1-\gamma |+\gamma ∥CA^{2}∥O\left(\lambda^{-1}\right)\right)∥\Delta u_{k}∥{}_{\lambda }. \end{array}$$

Since |1 − γ| < 1, there exists δ > 0 such that |1 − γ| + δ < 1. By selecting a sufficiently large λ, the following inequality can be satisfied

$$\begin{array}{l} \gamma ∥CA^{2}∥O\left(\lambda^{-1}\right)<\delta . \end{array}$$

There convergence of ||Δ*u*_*k*_||_λ_, i.e., Δ*u*_*k*_, has been proven. According to the convergence of Δ*u*_*k*_ and the inequality (26), it is obvious that $\underset{k\to \infty }{\lim}\Delta X_{k}=0$. Since *e*_*k*_(*t*) = *C*Δ*X*_*k*_(*t*), the convergence of *e*_*k*_(*t*), *t* ∈ [0, *T*], can be obtained immediately. □

## 5. Simulation and Experiment

In order to verify the effectiveness of the proposed ILC scheme, both simulations and experiments are conducted in this section.

### 5.1. System Identification

To evaluate the performance of the proposed method, a preliminary evaluation through simulation is conducted so as to reduce the cost of the experiment as well as expedite the experimental process.

The previous studies of the DEA modeling are generally based on the theory of DE material (Zhu et al., 2010; Li et al., 2013; Gu et al., 2017). However, the results of these studies can not totally satisfy the control purpose because the model is described in a set of differential equations and it is quite difficult to be used in model-based controller design (which can make the controller too complex). In this subsection, a data-driven method is employed to identified the knowledge-based model the DEA as mentioned in section 3.

**Figure 8:**
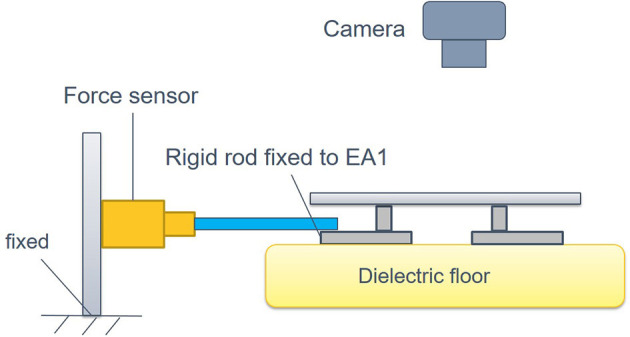
The experiment setup for system identification. Force sensor is used to measure the magnitude of the *F*_*a*_*ctive*, and the camera is employed to record the displacement.

To obtain the specific parameter values in the framework by means of system identification, the experimental setup as shown in Figure 8 has been developed, which consists of a EA foot (*EA*_3_) fixed with a force sensor while the others are free, and a camera used to record the change of displacement between the feet *EA*_3_ and *EA*_1_.

**Figure 9:**
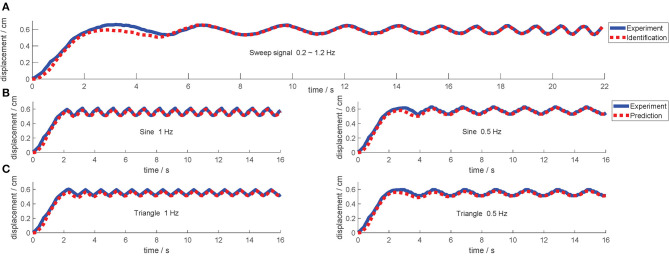
The result of the model identification and validation. The solid line is the response output of the actual model, and the dashed line is the response output of the identified model. **(A)** The identification result applying a sweep voltage signal. **(B,C)** Are validation results applying sine voltage signal and triangle voltage signals, respectively.

During the identification experiment, the DEA is driven by an identification signal that is a sinusoidal voltage sweep signal with the frequencies from 0.2 to 1 Hz, the amplitude of 0.36 kV and the offset voltage of 3 kV (the minimum actuate voltage is 1.8 kv). Then, the MATLAB System Identification Toolbox is used to estimate the parameters of the dynamic model. The identification result is shown in Figure 9A. The identified parameters are listed in Table 1. In addition, the equivalent mass $\tilde{m}$ is 0.12 kg and the frictional resistance force ζ is measured to be $0.1\tilde{m}g$, where *g* presents the gravity acceleration.

**Table 1:** The identified model parameters.

$\tilde{m}\left(kg\right)$		0.12	
ζ(*N*)		$0.1\tilde{m}g$	
*k*(*N* · *cm*^−1^)		3.456	
α(*N* · *cm*^−^1 · *kV*^−2^)		3.6	
β(*N* · *kV*^−2^)		-0.25	
*k_1_*(*N* · *cm*^−1^)	34.64	*c_1_*(*N* · *cm* · *s*^−1^)	0.4
*k_2_*(*N* · *cm*^−1^)	15.2	*c_2_*(*N* · *cm* · *s*^−1^)	5.067
*k_3_*(*N* · *cm*^−1^)	0.0396	*c_3_*(*N* · *cm* · *s*^−1^)	12

It is also important to emphasize that the robot in this study has obvious creep property, which is a property described by the viscoelasticity subjected to changes in load response over time. The creep property is mainly manifested as: the output deformation of the material with the constant load increase gradually as time goes by. The trend is briefly that the deformation initially increases rapidly in a short time, then gradually slows down with time passing by, and finally maintains stable. The creep response can be described by a different set of time constants, which in this model are primarily simulated by *c*_*h*_/*k*_*h*_ (*h* = 1, 2, 3). Therefore, another conditional constraint occurs in the identification process when considering creep characteristics: the time constants should be incremented step by step. In this way, the representation should comply with *c*_1_/*k*_1_ < *c*_2_/*k*_2_ < *c*_3_/*k*_3_. It can be found from Table 1 that the final adjusted parameters satisfy the above constraints. Consequently the identified model meets the constraints under “viscoelasticity,” which means that it has a certain physical property mapping.

After the identification, several signals with different forms (sine waves and triangle waves) and different frequencies are employed to validate the identified model, and the results are shown in Figures 9B,C.

As can be observed from the model validation results, the model simulated outputs can roughly match the actual system outputs and accordingly the creep under different voltage signals can be well predicted by this model. However, the simulated outputs do not 100% match the actual output exactly (according to the measured data, the fits between the model and actual system outputs mentioned above are all greater than 85%). More specifically, there is always a slight lag in the initial stage of the response. There are mainly two reasons: (i) there is a deviation in the model identification process, especially for the linearization operation applied to the actual model. After all, the identification process has not been done very accurately, it is just a simplified model. (ii) The intrinsic viscoelasticity with other properties of the DE material will cause some model uncertainties that cannot be predicted and eliminated during the modeling process. Therefore, this deviation cannot be eliminated by barely model parameter adjustment. Such deviation can be considered as part of the model uncertainties. This further embodies the importance of the control system, which can be solved by using a robust controller to compensate the uncertainties.

Obviously, the model developed in this paper is just a simplified version compared to the actual model of the DEA. The reasons why this simplified model is still acceptable in this study are as follows: (i) this is just a comparative simulation model, while its accuracy does not have much effect on the actual application (ILC works on iterative learning from zero or even unknown, instead of referring to the parameters under identified model); (ii) the excessive pursuit of model accuracy will extremely increase the model complexity and the cost, which is not the main focus in this work.

### 5.2. Simulation Results

After system identification, a mathematic dynamic model has been explained clearly which will then be used in simulation as the object model. Before simulating, it is necessary to emphasize here that the amplitude of the trajectory should be limited considering the displacement limitation with the physical model (mainly subject to hardware limitation with the characteristic of a high-voltage-induced breakdown, which is expected to be avoided with the method of voltage-limitation, equally to displacement-limitation). Thus, in order to maintain the consistency of the simulation with the actual situation (for subsequent physical verification), only the trajectories with the limited displacement (less than 2 cm) are appropriate to be given in this paper. Further more, the desired motion trajectory is not commonly a cumulative displacement trajectory, but a momentary displacement trajectory, that is, a single step length under the robot's movement at the current moment.

The parameters used in the simulation are given in Table 1. According to the dynamic model and the proposed controller, a simple trajectory (28) is first defined to verify the capability of the scheme, which only contains the “rising” curve. Significantly, in the design process, a smooth trajectory is discussed to be necessary for preventing the problems such as “high voltage breakdown” during the entire motion when the actual motion conditions are considered.

(28) $$y_{d,S}\left(t\right)=\{\begin{array}{ll} \frac{3t^{2}}{2000} & \left(0\leq t\leq 4\right)\\ -\frac{3{\left(t-20\right)}^{2}}{8000}+0.12 & \left(4<t\leq 20\right)\\ 0.12 & \left(t>20\right) \end{array},$$

**Figure 10:**
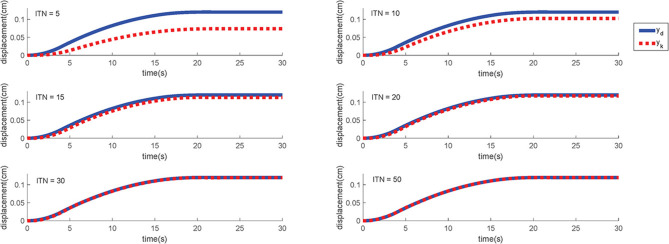
The simulation results of S-type signal under different iterations. ITN indicates the iteration numbers.

The initial input signals are set as *u*_0_ = 0 and γ = 0.05. The simulation result on the this simple trajectory is shown in Figure 10. As can be seen, the ILC control scheme can eventually track the expected trajectory well. More specifically, its actual motion trajectory gradually approaches to the target trajectory with the number of iterations increases. After 50 iterations, the output motion trajectory almost completely reaches the desired motion trajectory.

**Figure 11:**
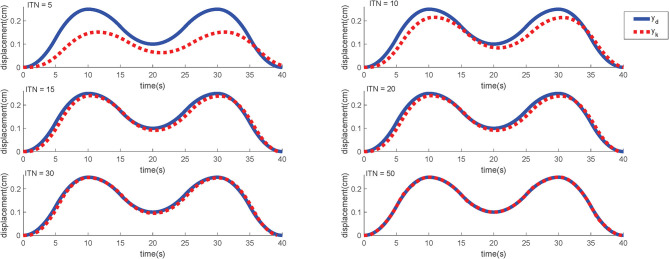
The simulation results of M-type signal under different iterations. ITN indicates the iteration numbers.

In order to verify the generalization of the proposed ILC scheme, another simulation is carried out. The target motion trajectory is defined as (29) and Figure 11 shows the simulation result. This trajectory is undoubtedly more complicated because it contains more basic curves such as “rising,” “falling,” “turning,” “peak,” “trough,” “regress.” In this way, the tracking effect of this trajectory can reflect the expected effect of almost all the remaining common curves, which makes it more representative.

(29) $$y_{d,M}\left(t\right)=\{\begin{array}{ll} \frac{t^{2}}{200} & \left(0\leq t\leq 5\right)\\ -\frac{{\left(t-10\right)}^{2}}{200}+0.25 & \left(5<t\leq 10\right)\\ -3\frac{{\left(t-10\right)}^{2}}{1000}+0.25 & \left(10<t\leq 15\right)\\ 3\frac{{\left(t-20\right)}^{2}}{1000}+0.1 & \left(15<t\leq 25\right)\\ -3\frac{{\left(t-30\right)}^{2}}{1000}+0.25 & \left(25<t\leq 30\right)\\ -\frac{{\left(t-30\right)}^{2}}{200}+0.25 & \left(30<t\leq 35\right)\\ \frac{{\left(t-40\right)}^{2}}{200} & \left(35<t\leq 40\right)\\ 0 & \left(t>40\right) \end{array},$$

In this simulation, the initial input signals are likewise set to *u*_0_ = 0 and γ = 0.05. As shown in Figure 11, the output of the control system can also substantially follow the desired motion trajectory very well after 50 iterations.

For specific simulation data, the MAER illustrates the convergence speed wand convergence accuracy in Table 2. After 30 iterations, the MAER can basically shrink to less than 2%, which shows a great efficiency.

**Table 2:** The convergence effect during the simulation.

ITN^*^		**5%**	**10%**	**15%**	**20%**	**30%**	**50%**
MAER^**^	*S*−*type*	47.53	18.75	7.39	3.76	1.11	0.51
	*M*−*type*	52.28	24.78	13.26	7.17	1.78	1.42
	*Experiment*	75.26	49.52	29.18	9.82		

* *ITN indicates the iteration numbers*.

** *MAER means Maximum Absolute Error Rate, its expression is $MAER=\frac{e_{max}}{y_{dmax}}$. e_max_ means the max error in the current iteration, y_dmax_ presents the max displacement in the desired trajectory*.

As can be discovered from the above results, benefiting from the iterative optimization process of ILC, the control effect (trajectory tracking performance) will become better and better with the continuous repetitive experiments. Thus, under enough iterations, the performance with the ILC will undoubtedly be more excellent than other common controllers as they would remain the same response during the repetitive situation.

In summary, the simulation results indicate that the proposed ILC scheme performs well on the motion trajectory tracking of the soft robot.

### 5.3. Experimental Results

To verify the effectiveness and the feasibility of the proposed ILC scheme in the real-time application, experiments of motion trajectory tracking are conducted in a wooden desk with the size of 1.8 × 2 m. The experimental platform is the soft robot detailed in section 2. Each EA is actuated by an external voltage amplifier (EMCO Q101-5) which can generate a maximum voltage of 10 kV. The DEA is actuated by an adjustable amplifier (Dongwen) which could be controlled by a micro-controller. Furthermore, an embedded micro-controller (Arduino UNO) is used to receive the external sensor signals and calculate the specific execution steps to control the specific actions of the robot through internal procedures. In addition, an Opti-tracking system is adopted to capture the motion of the robot precisely as well as get accurate acceleration errors ë at every moment.

The proposed ILC controller is model-free and the feedback information is off-line. However, the comparison between the model-free control schemes can hardly reflect the specific performance quantitatively. Hence, there is no comparative analysis between different control schemes in this study.

Considering the cost of time and the overlapping between the above two trajectories, we only apply the more complex “M-shaped” trajectory in the experiment.

**Figure 12:**
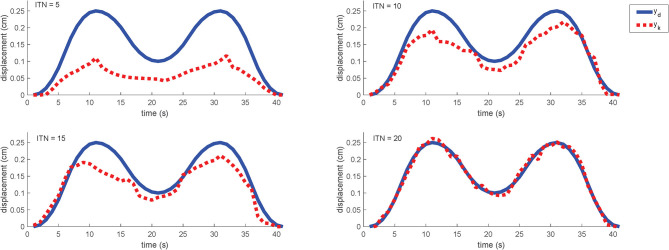
Motion trajectory profiles in different iteration cycle and ITN indicates the iteration numbers. Sampling time is set as 1 s, and the iteration period is 40 s.

Similar to the simulation, the desired trajectory is given by (29) and the learning gain is set as γ = 1. The initial control input as the zeroth iteration is the same as that in the simulation. Figure 12 shows the learning performance in each iteration cycle. One point should be mention here that the actual input is the voltage while the final output is displacement. Since that the voltage curves have a great relationship with the displacement curves according to the analysis in the section 3, the voltage curves would not be characterized for the sake of brevity.

As can be found from the experimental results, the motion trajectory at the first iteration has a very large tracking error from the desired trajectory. After that, the tracking error is gradually reduced under the control of the learning controller cycle-by-cycle and it can be almost eliminated to be zero after 20 iterations (the MAER is within 10% referring to Table 2). For the whole iterating time, it takes about 10 min to train a perfect control solution out without repetition. And for more details, we could find out that the overall trend with the motion is always consistent to the desired trajectory, while the optimization progress is strengthened steadily. As an online-adjusting with model-free controller (Tan, 2003), the ILC performs really good compared with other typical controllers in the consideration of adaptability and complexity. Hence, the experimental results also reveal that the proposed ILC scheme is effective for the motion trajectory tracking of the soft robot, equally it can help the system to achieve pretty good tracking performance.

## 6. Conclusion

In this study, an ILC method is proposed and applied to a DEA-based circular soft crawling robot in real-time which can achieve precise motion trajectory tracking performance. Both the simulation results and experimental results verify that the effectiveness of the ILC for the motion control of DEA-based soft robots. The main work of this study can be summarized as follows.

- According to the feature of DE materials, an electrically driven soft crawling robot combining the DEA with EAs referring to Qin et al. (2018) is built. It has the advantages of omnidirectional motion and periodic motion mode. This is a kind of minimalist robot unit, which can be assembled with other components in the future to realize some more complex functional robots. Furthermore, with the isotropy of the circular structure, the analysis of motion characteristics can be greatly simplified.
- A knowledge-based model framework, consisting of a series of spring-dashpot sets that usually employed to simulate the model of biological muscles, is used due to the similarities between the DEA and the biological muscle. In this way, the model of the DEA not only contains the priori knowledge but also simplify the representation of the practical DEA physical model, which can reduce the difficulty of the controller design.
- A partially model-free controller: ILC is employed for the motion trajectory tracking control, which can eliminate the difficulty of the accurate modeling. The periodic motion mode of the robot is fully conformed to the applicable range of the ILC controller. It is essential that the ILC scheme can help to compensate the uncertainties adaptively.
- Both simulations and experiments are conducted to verify the effectiveness of the developed soft robot and the proposed control scheme. The results show that the ILC scheme can help the robot to achieve excellent motion trajectory tracking performance in the case of the imperfect model.

## Data Availability Statement

The raw data supporting the conclusions of this manuscript will be made available by the authors, without undue reservation, to any qualified researcher.

## Author Contributions

All authors contributed to the manuscript drafting and writing. HC developed the robot prototype, designed the controller, conducted the simulations, and experiments. XL designed and analyzed the controller. WL and JC provided the guidance and support on the system design, modeling, and integration. QR conceived and supervised this research study.

### Conflict of Interest

The authors declare that the research was conducted in the absence of any commercial or financial relationships that could be construed as a potential conflict of interest.
